# Endogenous Retroviruses in Nervous System Disorders

**DOI:** 10.3390/ph14010070

**Published:** 2021-01-16

**Authors:** Victoria Gröger, Alexander Emmer, Martin S. Staege, Holger Cynis

**Affiliations:** 1 Department of Drug Design and Target Validation, Fraunhofer Institute for Cell Therapy and Immunology, 06120 Halle (Saale), Germany; victoria.groeger@izi.fraunhofer.de; 2 Department of Neurology, Martin Luther University Halle-Wittenberg, 06120 Halle (Saale), Germany; alexander.emmer@uk-halle.de; 3 Department of Surgical and Conservative Pediatrics and Adolescent Medicine, Martin Luther University Halle-Wittenberg, 06120 Halle (Saale), Germany

**Keywords:** human endogenous retrovirus, multiple sclerosis, amyotrophic lateral sclerosis, monoclonal antibodies

## Abstract

Human endogenous retroviruses (HERV) have been implicated in the pathogenesis of several nervous system disorders including multiple sclerosis and amyotrophic lateral sclerosis. The toxicity of HERV-derived RNAs and proteins for neuronal cells has been demonstrated. The involvement of HERV in the pathogenesis of currently incurable diseases might offer new treatment strategies based on the inhibition of HERV activities by small molecules or therapeutic antibodies.

## 1. Human Endogenous Retroviruses

Approximately 8% of human genomic DNA has high sequence similarity to retroviruses. These so-called human endogenous retroviral elements (HERV) are derived from exogenous retroviruses that have infected germ-line cells during evolution [1]. Once fixed in the population, these genetic elements were inherited as stable genetic components [1]. Some HERV display very high copy numbers, which might be the result of multiple germ line infections or reverse transcriptase-dependent amplification (retro-transposition) [2,3]. A phenomenon common to most HERV families, and particularly evident in HERV-K (HML-2), is their polymorphic nature, meaning that not all individuals have the same set of retroviruses at the same genomic sites. [4,5,6]. Such unfixed proviruses likely arose from divergence of retroviral copies, de novo insertions in the human population, or variable deletion of chromosomes [4,5,6]. As an example, HERV-K113 is present in a maximum of 30% of all individuals, showing a widespread geographic and racial variation [7,8].

Based on their similarity to exogenous viruses, the genomic structures of HERV possess the three viral genes *gag* (group specific antigens, encodes internal structural proteins), *pol* (encodes viral enzymes), and *env* (encodes the envelope protein), which are flanked by regulatory long terminal repeats (LTRs). Additional ERV-derived proteins, which are products of alternative splicing, include the regulatory rec and np9 proteins. However, most of the ERV open reading frames are mutated and cannot produce functional proteins or virions [9]. An earlier genome-wide search revealed only 29 *env*, 17 *gag*, and 13 *pol* open reading frames (ORF) longer than 500 codons, which possibly code for viral proteins, among a total of 38,000 retroviral ORFs examined [10]. The maintenance of ORFs in HERV genomes over many thousands of years of evolution suggests a functional role for these elements. However, an intact ORF alone is not sufficient for protein expression, since HERV are usually epigenetically silenced. Only if HERV become reactivated by intrinsic or extrinsic factors, can viral RNAs and proteins be produced. Their function is largely unknown, even though understanding of their importance has increased in recent years. Both the beneficial and detrimental effects of encoded viral proteins have been reported. Participation in normal physiological processes, such as placental development [11] and modulation of innate immunity [12], shall be mentioned here as examples. Independent of their protein-coding capacity, HERV are able to regulate neighboring genes by providing alternative promoters [13] or by altering the chromatin structure by binding co-repressor proteins like TRIM28 [14,15].

Emerging evidence suggests that members of the HERV-K, HERV-W, and HERV-H families have the potential to regulate immune function [16,17,18,19]. Hence, their aberrant expression has been linked to the development and progression of inflammatory and neurologic diseases, although causal links have yet to be established. In the present review, we will focus on the current state of knowledge on the association of HERV with multiple sclerosis (MS), amyotrophic lateral sclerosis (ALS), and other nervous system disorders. Additionally, the potential of HERV as new therapeutic targets will be highlighted. First, however, a general overview is given of how HERV sequences can be reactivated, as this is a basic prerequisite for possible pathogenic effects.

## 2. Regulation of HERV Expression

To ensure genomic stability and integrity, HERV are usually transcriptionally silent. This is accomplished by DNA methylation and histone modifications [20,21,22,23]. The majority of endogenous retroviral sequences are located in chromosomal regions with repressive, heterochromatic chromatin architecture leading to low transcriptional activity in most cell types [21]. This “epigenetic corset” is established during embryogenesis. However, in early embryonic stages, which are characterized by global hypomethylation, precise regulation of retroviral sequences seems to be involved in physiologic processes such as the induction of viral restriction pathways [24] and the differentiation of stem cells. For instance, neural differentiation involves tight control of HERV-H RNAs via death-associated protein 5 (DAP5, also known as novel APOBEC-1 target 1, NAT1) and the terminal uridyltransferase TUT7 [25]. Similarly, the down-regulation of highly expressed HERV-K (HML-2) envelope protein in pluripotent stem cells results in dissociation of the stem cell colonies and increased differentiation along neuronal pathways [26].

If the epigenetic control machinery becomes impaired, endogenous retroviral sequences can be activated and become transcriptionally active [27,28]. This is particularly evident in cancer because DNA methylation in cancer cells is often severely impaired. As a consequence, the activity of many HERV, particularly HERV-K, is frequently elevated in tumors like melanoma, breast cancer, and astrocytoma (reviewed in [29]). In contrast, development-specific demethylation in placental tissue leads to the physiologically required expression of HERV during placentogenesis. Syncytin-1, the envelope protein of the HERV-W family member HERVWE1, was shown to contribute to the formation of the syncytiotrophoblast by its membrane fusogenic capacity and seems to also be involved in maternal immune tolerance towards the fetus [30,31].

Additional to epigenetic mechanisms, environmental factors such as caffeine and aspirin are supposed to be regulators of HERV expression [32], although in vivo evidence for this is still lacking. In particular, infections with exogenous viruses represent potent triggers of HERV activation. Thus, transactivation of HERV by human immunodeficiency virus 1 (HIV-1), hepatitis B virus (HBV), human T-lymphotropic virus 1 (HTLV-1), and influenza A virus has been described [33,34,35,36]. For example, the HIV-1 transactivator of transcription (Tat) protein can induce the expression of HERV in lymphocytes and astrocytes through regulation of the nuclear factor kappa B (NFκB) pathway, the nuclear factor of activated T cells (NFAT) pathway, and the toll-like receptor 4 (TLR4) pathway [37,38]. In accordance with that, HIV-1 infected patients show increased antibody titers against the transmembrane unit of HERV-K (HML-2) envelope protein, which decrease with antiviral treatment [39]. The transactivator protein Tax of HTLV-1 increases, similar to HIV-1 Tat, the promoter activity of HERV-K, HERV-W, HERV-H, and HERV-E members in T cells [35]. Herpesviruses including Epstein–Barr virus (EBV) [18,40,41], herpes simplex virus 1 (HSV-1) [42,43,44], and human cytomegalovirus [45] have also been reported to induce HERV transactivation. As a probably important example, the EBV glycoprotein gp350 triggers transcription of the HERV-K18 *env* gene in resting B cells after binding to the EBV receptor CD21 [41]. The same gp350 stimulates HERV-W Env protein expression in astrocytes through the NFκB pathway [46]. Interestingly, since EBV infection is an important risk factor for the development of MS, HERV are discussed as the missing link between EBV infection and disease onset [47]. Additional information on the virus-associated regulation of HERV elements is provided in a recent review by Chen et al. [48]. Infections with other pathogens including *Toxoplasma gondii* were shown to induce a wide range of HERV elements in the Ewing sarcoma cell line SK-N-MC [49]. Inflammatory conditions per se, such as interferon gamma (IFNγ) and other proinflammatory cytokines, were shown to induce HERV expression in vitro [50,51]. It is not yet clear whether HERV occur as a consequence or as a cause of inflammatory processes. For this reason, the interaction of HERV with the immune system is being intensively investigated, especially in the context of diseases such as multiple sclerosis.

## 3. Multiple Sclerosis

Multiple sclerosis (MS) is a chronic inflammatory disease of the central nervous system resulting in progressive neurodegeneration and neurological disability. The onset usually occurs between the ages of 20 and 40 and is often evidenced by a clinically isolated syndrome (CIS), i.e., a first episode of neurological symptoms caused by inflammation or demyelination [52]. The type of symptomatology is diverse and depends on the affected central nervous system regions; common early signs include vision problems, weakness or fatigue, and balance problems. MS can typically be divided into three clinical types: (i) Relapsing-remitting MS (RRMS), which is characterized by discrete relapses with intermediate periods of remission. Relapses manifest themselves in new or worsening symptoms with underlying active brain lesions with lymphocytic inflammation. (ii) Secondary progressive MS (SPMS) typically develops 10–15 years after RRMS onset, characterized by a slowly progressive disease course with dominant neurodegeneration. (iii) Primary progressive MS (PPMS) is characterized by gradually increasing disability from the onset of the disease involving a dominant neural system, such as lower limb weakness and spasticity; 5–15% of MS patients show a PPMS onset.

White matter lesions incidentally found by magnetic resonance imaging (MRI) in individuals without a clinical history of demyelinating attacks or any other cause of white matter lesions (radiologically isolated syndrome, RIS) indicate that MS begins before the first clinical symptoms become apparent [53,54]. The early clinical course is marked by relapses from which symptomatic recovery is usually complete. Transition from RRMS to SPMS is subtle with relapses occurring on a low background level of progression, before progression becomes dominant. This gradual clinical disease development is consistent with rather continuous pathological changes. At the beginning of the disease, inflammation is suggested as the driving force, while the progressive phase is dominated by neurodegenerative processes. Nevertheless, cognitive impairment and progressive MRI-detectable atrophy occur in early MS, suggesting that neurodegeneration is already present from clinical onset.

The pathological hallmarks of MS are focal inflammatory lesions characterized by primary demyelination in the white and grey matter of the CNS. Oligodendrocyte damage and demyelination originate in the periphery with the activation of self-reactive T cells that infiltrate in the CNS across a disintegrating blood–brain barrier [55]. In active lesions, activated microglia or macrophages, mainly CD8-positive cytotoxic T-lymphocytes, and fewer CD4-positive helper T cells, B cells, and plasma cells can be found [56]. The inflammatory process is accompanied by the activation of astrocytes leading to the formation of astroglial scars [55,57]. Acute axonal damage is most prominent in the early stages of RRMS and SPMS, whereas in PPMS, axon degradation is more constant [58]. Whether axonal damage occurs as a consequence or independently of demyelination is subject to controversial discussion [59]. 

Extensive remyelination can be frequently observed during the early stages of RRMS [60]. However, recurrent inflammatory attacks and the failure of myelin repair during later progressive phases of the disease ultimately lead to permanent de-myelinization. There is currently no causal therapy for MS. Treatment is designed to reduce inflammatory processes and prevent the progression of symptoms.

Although the cause of MS remains unclear, it is believed that a combination of genetic and environmental factors influences disease susceptibility. Studies with siblings of affected individuals and monozygotic twins indicate a strong genetic component [61,62]. The human major histocompatibility complex (MHC) region on chromosome 6p21 was identified early as the strongest genetic locus for MS [63,64]. More recent genome-wide association studies uncovered more than 200 implicated genetic risk variants, including 110 non-MHC genetic loci [65,66,67], which all confer small increases in disease risk. Moreover, genetic differences between RRMS and PPMS have been identified [68]. The association of different HERV loci and MS risk will be discussed below. The multifactorial character of MS becomes particularly evident in studies showing that the individual risk of disease in genetically predisposed individuals increases when they are additionally exposed to environmental risk factors [69,70,71,72]. Such factors include low vitamin D levels, lack of sun exposure, female sex, EBV infection, obesity during adolescence, and smoking [69]. Among these factors, infection with EBV is considered the strongest risk factor, as the risk of developing MS increases 15-fold with EBV infection in childhood and 30-fold with infection in adolescence compared with uninfected individuals [73]. Moreover, there is a higher frequency of EBV seropositivity in MS patients compared to controls [74] and the beneficial effects of EBV-specific T cell therapy in MS have been demonstrated [75]. Several mechanistic hypotheses addressing the etiologic role of EBV in MS exist and are summarized in a recent review [76].

### 3.1. HERV-W in MS

In line with a possible virus-associated etiology of MS, the involvement of HERV represents an additional piece of the puzzle in this multifactorial and heterogeneous disease. Over the past 30 years, concurrent studies by several investigators have consistently suggested a relationship between HERV and the development of MS.

The first observation of retroviral particles of presumed endogenous origin in MS patients dates back to the early 1990s [77]. The cDNA sequences derived from particle-associated RNA were then assigned to a “multiple sclerosis-associated retrovirus” (MSRV). Merely a decade later, it was discovered that MSRV belongs to the HERV-W family [78]. HERV-W is a multicopy family with about 650 loci in the human genome [79]. Most of these loci are coding-deficient due to their evolutionary age [80]. However, more than 100 HERV-W loci were found to be transcribed in the human brain [79], although it cannot be excluded that some of these transcripts are caused by recombination events in vitro [81]. Other investigators identified seven transcribed HERV-W env loci in peripheral blood mononuclear cells (PBMC) [82]. Among 13 reported HERV-W loci with full-length env genes, only the HERVWE1 locus on chromosome 7q21.2 codes for a complete HERV-W envelope protein called syncytin-1 [82]. ERVWE2 on chromosome Xq22.3 encodes an incomplete HERV-W env of unknown function [83]. Since there is no counterpart to the initially named MSRV envelope protein (GenBank sequence AF331500) in the human genome, the origin of MSRV env remains open [82,84]. Based on the assumption that reverse transcriptase can switch templates during in vitro PCR amplification [81], recombination of different HERV-W env loci transcripts was proposed [82]. The origin of different published MSRV sequences involving recombination of transcripts from up to six different HERV-W loci is discussed in more detail by Grandi et al. [84]. Other possible explanations for the discrepancy in HERV-W genomic sequences found by various investigators include unfixed copies of HERV-W that are present only in a certain percentage of the population, and the occurrence of somatic recombination events that cannot be detected in unaffected cells [81,85,86].

HERV-W proteins have been found to be physiologically expressed in the normal brain with unknown function [87,88]. Increased amounts of HERV-W RNA, DNA, proteins, virions, and antibodies directed against HERV-W peptides in the blood, cerebrospinal fluid (CSF), and/or brain of MS patients have been associated with disease etiology. HERV-W env has been detected in the brains of MS patients, particularly in macrophages and microglia in lesions, but not in healthy controls [89]. Similarly, active MS lesions show an accumulation of HERV-W gag in axonal structures and endothelial cells as well as specific expression of HERV-W env in macrophages and microglia cells [87,89,90,91]. Additionally, HERV-W env is elevated on the surface of B cells and monocytes [92,93,94]. Concerning seroreactivity, higher antibody titers against HERV-W env have been identified in patients with active MS compared to patients with stable MS [92], patients with neuromyelitis optica spectrum disorder [95,96], or healthy controls [97,98].

There are several studies that report increased copy numbers of HERV-W pol and env DNA or RNA in the blood (PBMC or serum/plasma) and/or CSF in patients with MS compared to control groups (healthy controls or patients with other neurological diseases) [90,99,100,101,102,103,104]. In this context, the presence of the MRSV in the CSF seems to be associated with disability accumulation and a higher rate of relapses in MS patients in a 10-year follow-up study in a Sardinian cohort [105]. Interestingly, one study found an inverse correlation between MSRV DNA copy numbers and vitamin D concentration in RRMS patients [106]. Although all mentioned studies report increased HERV-W viral loads when comparing MS patients with healthy controls, not all MS patients tested positive for HERV-W/MSRV RNA or anti-HERV-W env antibodies [95,100,107]. Consequently, the detection of HERV-W alone is not sufficient to distinguish MS patients from healthy individuals or patients with other neurological disorders. The variable expression of HERV in MS patients may rather reflect a differential regulation of inherited HERV copies in the genome. Thus, more detailed studies are still needed to determine the applicability of HERV-W as a diagnostic marker in MS.

The hypothesis that HERV-W is a driving factor in the development of MS is further challenged by studies reporting no association between HERV-W yields and the disease. For example, the relative transcript levels of investigated HERV-W elements in PBMC or the brain did not differ significantly between MS patients and controls [79,82] and no HERV-W was detected in the cerebrospinal fluid of MS patients using PCR [108]. Ruprecht and colleagues reported the absence of antibodies and T-cell reactivity against MSRV env and gag proteins in MS patients, respectively [109]. This might be due to self-tolerance to HERV as autoantigens in the investigated subjects. The positive seroreactivity against HERV-W mentioned above [92,95,96,97,98] referred to specific HERV-W peptides being antigenic and leading to a rather low response amplitude, which is consistent with variations in “natural autoantibodies” when corresponding tolerated antigens are released (cell death, tissue damage) or abnormally expressed (HERVs) [110]. Therefore, Ruprecht’s observations do not contradict the other studies. Another study reports syncytin-1 encoding RNA to be increased in MS brains compared to non-MS patients, but not in CSF and plasma [111]. Most of these studies do not consider the treatment effects on HERV formation and persistence. It was shown that interferon-beta (IFN-β) treatment reduces MSRV levels in the plasma of MS patients below detection limits after three months of treatment [112]. Likewise, elevated antibody titers against HERV-W env in MS patients decreased after IFN-β treatment [97]. This may explain in part the absence of HERV-W in other investigations. However, the complex genomic distribution of HERV-W elements leads to limitations in the comparability of studies, since it often remains unclear from which genetic locus the investigated protein or nucleic acid originates. Despite these limitations, a meta-analysis of 12 studies performed by Morandi and colleagues reports a strong association between MSRV/HERV-W pol and env and MS [104].

Although it is not known whether HERV-W has a causal role in the development of MS, there is some evidence that it interacts with the immune system. Thus, MSRV/HERV-W env protein induces the release of inducible nitric oxide synthase and pro-inflammatory cytokines such as tumor necrosis factor alpha, IFNγ, interleukin (IL)-6, and IL-1β from PBMC of MS patients [16,113,114,115]. Moreover, IL-6 and the p40 subunit of IL-12 seem to correlate with disease severity [113]. In addition, the activation of certain cytokines by HERV-W env depends on the clinical course of the patient’s disease. For example, so-called type I cytokines (which favor cellular immune responses) predominate in stimulated PBMC of patients with acute MS, while in patients with stable MS, a type II cytokine profile (which favors humoral immune responses) dominates [116,117]. Interestingly, combined stimulation of PBMC from MS patients with antigens from *Herpesviridae* (herpes zoster virus, human herpesvirus 6A, herpes simplex virus 1) and antigens from HERV-H also leads to an altered Th1/Th2 response [114]. 

It has been shown for human brain endothelial cells, rat and human oligodendroglial precursor cells, and human PBMC that the HERV-W-induced release of proinflammatory cytokines is mediated by concentration-dependent activation of the pattern recognition receptors CD14 or TLR4 [16,113,115,118,119].

Increased cytokine release after MSRV/HERV-W env stimulation has also been recorded in human brain endothelial cells and rat oligodendrocyte precursor cells (OPCs) [118,119]. In addition to altered cytokine profiles, rat OPCs show a decrease in myelin proteins CNP and MBP as opposed to the unchanged expression of the precursor marker claudin 11 (CLDN11, also known as O4 antigen) following HERV-W env stimulation. This reflects the impairment of myelin sheath formation and cellular differentiation caused by HERV-W env [119]. These negative effects can be successfully neutralized by the anti-HERV-W env antibody GNbAC1 [120,121]. Cytotoxicity on OPCs might also be mediated via HERV-W expression in astrocytes as it induces endoplasmic reticulum stress and the release of redox reactants leading to OPC apoptosis [122]. In accordance, transgenic mice overexpressing HERV-W showed neuroinflammation, decreased levels of myelin proteins in the corpus callosum, and behavioral deficits compared to wild type littermates [123]. In addition, MSRV/HERV-W env causes axonal injury by altering microglial cells to form a degenerative phenotype and to associate with myelinated axons [91]. The immuno-stimulatory properties of HERV-W env protein were demonstrated using a myelin oligodendrocyte glycoprotein-induced mouse model of experimental autoimmune encephalomyelitis (EAE), in which mice injected with HERV-W instead of mycobacterial lysate developed a disease phenotype [124].

In summary, MSRV/HERV-W env is expressed in chronic active MS lesions. Preclinical models have shown that HERV-W env is a negative regulator of OPC maturation and a TLR4 agonist that activates innate immunity and might be involved in MS etiology.

### 3.2. Other HERV in MS

In addition to the HERV-W family, an association of other HERV with MS, such as HERV-H/F and HERV-K, has been suggested.

Concerning the HERV-H/F family, increased MS risk in Danish and Norwegian populations was related to the single nucleotide polymorphism (SNP) rs391745 in the vicinity of the *HERVFC1* gene locus [125,126]. The same SNP showed significant association with MS in two Spanish cohorts [127]. Additionally, the amount of HERVFC1 gag RNA in plasma and of HERVFC1 gag protein in T cells and monocytes was increased in active MS patients compared with non-active MS and healthy controls [128]. Whereas HERV-H env and gag RNA was increased in the serum and PBMC of Danish MS patients [129,130], no difference in RNA expression (analyzed in CSF, PBMC, and brain) between MS and patients with other neurological diseases was observed in Spanish and Canadian studies [108,111,122,131]. Expression of HERV-H3 was shown to be significantly increased on the surface of non-classical monocytes in patients with CIS and RRMS compared to healthy controls [132]. However, expression of HERV-H3 seems to be inconsistent and probably depending on pre-treatment of patients [133] as immune-modulating therapy can lead to an increase of nonclassical monocyte populations [134].

Another HERV family implicated in MS is HERV-K (HML-2), which has many human-specific insertions as it contains many of the most recently integrated and “active” retroelements [135,136]. Studies analyzing HERV-K DNA, RNA, and protein levels in MS patients compared with healthy controls report different and sometimes inconsistent results depending on the member of the HERV-K family examined, the part of the virus analyzed (*env*, *pol*, or *gag*), and the investigated tissue. For example, expression of HERV-K *pol* [51], but not of HERV-K *env* [111,122], was elevated in the brain of MS patients compared to control groups. Moreover, an increase in the prevalence of HERV-K113, but not HERV-K115, was reported in patients with MS or Sjögren’s syndrome [7]. However, the same group failed to reproduce these findings in a larger cohort of MS patients [137]. The difference between these studies might be explained by the genetic variability between the populations studied [135,138]. From a genetic point of view, homozygous carriers of the HERV-K18.3 allele have a higher risk of developing MS compared with carriers of the other two alleles K18.1 or K18.2 [139,140]. Moreover, the SNP rs2435031 near the HERV-K113 locus has been shown to be associated with MS [125]. Based on published data, relevance for HERV-K in MS appears to be low. Instead, there is evidence of HERV-K involvement in amyotrophic lateral sclerosis (ALS).

## 4. HERV-K in Amyotrophic Lateral Sclerosis

ALS is a disease for which only symptomatic therapy is available. The etiology of ALS is unknown. Several mutations in seemingly unrelated genes, e.g., superoxide dismutase 1 (SOD1), chromosome 9 open reading frame 72 (C9orf72), or TAR DNA-binding protein 43 (TARDBP), have been described and it remains possible that ALS is not a single disease entity but a conglomerate of different diseases with a similar clinical endpoint. In most patients, however, no recurrent mutations can be found and for these “sporadic” ALS cases, non-genetic factors might play a role [141,142,143].

Epidemiological observations suggest that viruses might be involved in the disease. However, no exogenous virus has been found until now. Observations about ALS-like symptoms in patients with HTLV-1 infection remain anecdotal [144]. Reverse transcriptase (RT) activity is a hallmark of retrovirus infection. Early studies found elevated RT activity in the serum of patients with sporadic ALS [145]. Interestingly, the RT activity was not elevated in spouses of the ALS patients, but non-symptomatic blood relatives of ALS patients showed a similar elevation of RT activity to the ALS patients. This observation strongly suggests that the RT activity is not a consequence of infection with an exogenous virus but a consequence of genetic factors that influence the expression or activity of endogenous reverse transcriptase. Recently it has been shown that the human DNA polymerase eta has RT activity [146]. However, HERV are the best candidates for sources of the elevated RT activity in ALS patients. Indeed, high levels of HERV-K pol transcripts were found in the brain from ALS patients [147]. The expression correlated with the expression of the ALS-associated TARDBP and HERV-K pol protein co-localizes with TARDBP protein in neurons. HERV-K expression was not restricted to a single locus but, interestingly, a preference for *pol* loci with intact open reading frames was observed. Moreover, it was shown that HERV-K is expressed in the brain of ALS patients and that HERV-K can induce the apoptosis of neuronal cells in a mouse model [148]. In addition, antibodies with specificity for HERV-K have also been found to be elevated in patients with ALS [98].

TARDBP might play an important role in the activation of HERV-K. It was shown that the transgenic expression of mutated TARDBP leads to the accumulation of HERV-K proteins, especially reverse transcriptase [149]. Expression of TARDPB in *Drosophila* neuronal cells and glia cells induces the expression of diverse repetitive elements and leads to age-dependent neurodegeneration [150]. TARDPB inhibits the siRNA-mediated silencing of the *Drosophila* gypsy element (a *Drosophila* ERV) and in particular, gypsy seems to be responsible for the toxic effects on glia cells [150] and neuronal cells [151]. Interestingly, the toxicity of gypsy can spread to neighboring cells [151]. The activation of repetitive elements might be a common scheme of ALS-specific mutations. For instance, multiple repetitive elements are expressed in the brain of ALS patients with mutations in C9orf72 and the transgenic expression of C9orf72 in human embryonic kidney 293 cells leads to RNA polymerase II-dependent expression of repetitive elements [152]. Vice versa, it has been shown that knock-out of HERV-K env in prostate cancer cells results in the down-regulation of TARBP expression [153].

The reciprocal regulation of HERV and ALS-specific genes like TARBP requires further investigation. Stimulation with cytokines has also been shown to induce the expression of HERV-K in neuronal cells [154]. This poses the general question of whether HERV activation is an initializing step during disease development or whether HERV activation is a consequence of ongoing degeneration and inflammatory pathways. It should be noted that the involvement of HERV and especially HERV-K in ALS is discussed controversially. Some investigations failed to show increased HERV-K in ALS patients compared to controls [155,156]. Whether this can be explained by technical reasons solely remains an open question [157,158].

## 5. HERV in Other Nervous System Disorders

In addition to MS and ALS, other nervous system disorders have been associated with HERV activation. Elevated HERV transcripts have been found in the brains of patients with Alzheimer disease (AD) [159,160,161]. Interestingly, HERV-K RNA from the *env* region induces neurodegeneration in an TLR7- or TLR8-dependent manner [159]. TLR7 and TLR8 are two highly similar pattern recognition receptors that are activated by single-stranded RNA. The activation of these receptors by HERV-K RNA and subsequent neurodegeneration shows that HERV RNA can have profound biological effects without the necessity for HERV protein synthesis. Another interesting disease with possible involvement of non-coding RNAs from repetitive elements is Aicardi–Goutieres syndrome (AGS). AGS is caused by mutations in genes involved in the regulation of cytoplasmic nucleic acids and these mutations are considered to lead to the accumulation of double-stranded RNA, RNA/DNA hybrids, or single stranded DNA that activate innate immune response pathways (reviewed in [160]). The nucleic acids involved in the activation of innate immune responses in AGS are probably derived from repetitive elements of the L1 and Alu classes. However, HERV-derived sequences have also been found at least in animal models for AGS [161].

A hallmark of Alzheimer disease is the intraneuronal accumulation of pathogenic forms of the microtubule-associated protein tau. Interestingly, pathogenic tau can induce the expression of repetitive elements including HERV. In *Drosophila*, pathogenic tau induces the expression of gypsy and other transposable elements in an age-dependent manner [162,163]. One mechanism for this activation of ERV by tau seems to be the depletion of piwi-interacting RNAs (piRNAs). piRNAs are small non-coding RNAs that are known for their role in the repression of transposable elements, especially in the germline [164].

For other nervous system diseases, only a few studies have analyzed possible associations with HERV. Elevated expression of HERV has been found in progressive supranuclear palsy [163], chronic inflammatory demyelinating polyradiculoneuropathies [165], fibromyalgia [166], myalgic encephalomyelitis [167,168,169], prion disease [170,171,172], and spinal and bulbar muscular atrophy (SBMA) [173]. In the case of SBMA, HERV RNA was found unchanged but elevated levels of processed gag antigens have been found. This seems interesting as SBMA is caused by mutations in the androgen receptor (*AR*) gene. AR signaling has been shown to activate HERV LTRs and HERV-derived np9 protein has been shown to activate AR signaling [174]. Such reciprocal activation might be involved in the initiation of vicious cycles that freeze the activation status of signaling pathways. Interestingly, it has been reported that progerin, the protein constitutively expressed in patients with progeria but also increasingly expressed in aging normal individuals, inhibits the expression of transposable elements [175]. The author of this study speculates that progerin might serve as a safeguard which inhibits the aberrant activation of HERV and other transposable elements in aging cells [175]. With increasing age, the chance for stochastic activation of vicious cycles like the AR-np9 cycle increases, which might also explain the age-dependency of the aforementioned *Drosophila* models. The presence of safeguard mechanisms against such vicious cycles seem meaningful.

In addition to the mentioned neurological diseases, increased HERV expression has been described for some mental disorders, including autism spectrum disorders [176,177], attention deficit hyperactivity disorder [178,179,180], and schizophrenia. HERV-W and to a lower extent, HERV-K, have been found increased in the CSF and blood of patients with schizophrenia [181,182,183,184,185,186]. HERV-W env protein has been shown to alter glutamate synapse maturation in the developing brain, which might be an important factor for the development of neuropsychiatric disorders [187]. Moreover, polymorphisms in HERV loci or adjacent genes have been associated with schizophrenia [188,189,190,191,192,193]. One of these loci, proline dehydrogenase 1 (PRODH) from human chromosome 22, is an interesting example for the co-regulation of HERV and neighboring genes [193,194]. Here, the LTR form of an HERV (ERVK-24) drives the expression of HERV sequences as well as the expression of PRODH. Germ cell tumors with high expression of this HERV also show high expression of PRODH and forced differentiation of these tumor cells results in concomitant down-regulation of PRODH and ERVK-24 [194]. 

## 6. Possible Therapeutic Implications

With our current knowledge, HERV-W and HERV-K at least seem most likely to be involved in the pathogenesis of nervous system disorders, especially of MS and ALS. Therefore, new innovative therapeutic approaches are focusing on the reduction of HERV load in patients in order to achieve a positive effect on disease progression.

One approach assumes that highly active antiretroviral therapy (HAART), which is the standard treatment for HIV patients, blocks HERV expression. The experimental bases for this hypothesis are studies in HIV-positive patients, which showed a significant reduction in HERV-K RNA in plasma samples of successfully treated patients compared to patients who might have developed drug-resistance and/or received suboptimal therapeutic doses [195]. Other studies showed that the antibody titers against the transmembrane unit of HERV-K (HML-2) decreased with antiviral treatment [39]. 

Antiretroviral therapy has been applied both in ALS and MS. A pilot clinical trial of the protease inhibitor, indinavir, failed to show efficacy in ALS [196]. Interestingly, patients with HIV infection who subsequently developed motor neuron disease showed reduced HERV-K expression in blood following antiretroviral treatment. At the same time, the symptoms of three of these five patients regressed [197]. HIV-infected individuals with ALS might represent a unique sub-population of ALS patients that activate HERV under the influence of HIV. Therefore, anti-HIV drugs may turn off the activating effect of HIV on HERV. In ALS patients without HIV, alternative mechanisms might activate HERV. A phase 2a open label study with the antiretroviral drug Triumeq (which includes the two reverse transcriptase inhibitors abacavir and lamivudine, and the integrase inhibitor dolutegravir) has been conducted in 43 ALS patients (ClinicalTrials.gov Identifier: NCT02868580). Triumeq significantly decreased HERV-K env DNA in serum and showed a strong potential for biological activity as disease progression assessed on the ALS Functional Rating Scale was slowed down [198].

A case study on a patient with HIV infection and MS reported positive effects from antiretroviral treatment on MS symptoms [199]. In contrast, a phase 2a pilot study in 20 RRMS patients could not confirm this observation. In this study, patients showed no reduction in gadolinium-enhancing lesions on MRI after 3 months of treatment with raltegravir, an inhibitor of HIV integrase [200]. Since integrase-dependent chromosomal reintegration of viral DNA is not relevant for HERV transactivation, raltegravir may not have had an effect on HERV expression in MS, which may explain the negative results of the study. Furthermore, HERV might be transactivated by the HIV protein Tat [37]. Thus, it is possible that the effect of antiretroviral therapies on HERV is only indirectly based on the primary reduction of the HIV load and the concomitantly reduced transactivation of HERV. Thus, in patients without HIV, antiretroviral therapy would have no effect.

A completely different therapeutic approach assumes that harmful HERV proteins can be inactivated with specific antibodies. In this context, studies of the HERV-W-specific monoclonal antibody (mAb) GNbAC1, also called temelimab, are the most advanced. Temelimab is a recombinant, humanized IgG4κ mAb with high affinity (Kd = 2.2 nM) to the surface domain of a HERV-W envelope protein, for which a cDNA was cloned from virion-associated genomic RNA isolated from MS cell cultures [201]. In preclinical studies, temelimab could rescue myelin basic protein (MBP) expression in OPCs and improve the symptoms caused by HERV-W env in an EAE mouse model [120,121,202]. Three clinical phase 1 studies in a total of 78 healthy male volunteers showed good tolerability of intravenous-administered temelimab at doses up to 110 mg/kg without severe adverse drug reactions [203,204,205]. A rather small study on 10 MS patients confirmed the dose-linear pharmacokinetics and tolerability of previous studies [206,207]. The clinical phase 2b study CHANGE-MS (ClinicalTrials.gov Identifier: NCT02782858) and its extension ANGEL-MS (ClinicalTrials.gov Identifier: NCT03239860) were conducted in 270 and 219 RRMS patients, respectively [208]. According to publicly available information, the study did not show a significant reduction in the total number of gadolinium-enhancing lesions and clinically apparent relapses in temelimab-treated groups thereby failing to reach a primary study endpoint [209]. However, a benefit for brain atrophy and for the magnetization transfer ratio (MTR) signal of patients treated with the highest dose relative to the placebo group was claimed, suggesting a pharmacodynamic effect of temelimab in remyelination. These data await peer-reviewed publication. 

In conclusion, numerous studies suggest the expression of HERV in different neurologic disorders. Whether it happens as bystanders of the disease or even in a causative fashion is currently unclear. Therefore, future studies are required to elucidate the nature of contribution of HERV to these disorders.

## Data Availability

Data sharing not applicable.
